# Identification of a long non-coding RNA regulator of liver carcinoma cell survival

**DOI:** 10.1038/s41419-021-03453-w

**Published:** 2021-02-15

**Authors:** Yulia Rybakova, John T. Gonzalez, Roman Bogorad, Vikash P. Chauhan, Yize L. Dong, Charles A. Whittaker, Timofei Zatsepin, Victor Koteliansky, Daniel G. Anderson

**Affiliations:** 1 David H. Koch Institute for Integrative Cancer Research, Massachusetts Institute of Technology, Cambridge, MA 02142 USA; 2 Skolkovo Institute of Science and Technology, Moscow, 121205 Russia; 3 Department of Electrical Engineering and Computer Science, Massachusetts Institute of Technology, Cambridge, MA 02142 USA; 4 Department of Biology, Massachusetts Institute of Technology, Cambridge, MA 02142 USA; 5 Institute for Medical Engineering and Science, Massachusetts Institute of Technology, Cambridge, MA 02139 USA; 6 Harvard and MIT Division of Health Science and Technology, Massachusetts Institute of Technology, Cambridge, MA 02139 USA; 7 Department of Chemical Engineering, Massachusetts Institute of Technology, Cambridge, MA 02139 USA

**Keywords:** Cancer genetics, Oncogenes

## Abstract

Genomic studies have significantly improved our understanding of hepatocellular carcinoma (HCC) biology and have led to the discovery of multiple protein-coding genes driving hepatocarcinogenesis. In addition, these studies have identified thousands of new non-coding transcripts deregulated in HCC. We hypothesize that some of these transcripts may be involved in disease progression. Long non-coding RNAs are a large class of non-coding transcripts which participate in the regulation of virtually all cellular functions. However, a majority of lncRNAs remain dramatically understudied. Here, we applied a pooled shRNA-based screen to identify lncRNAs essential for HCC cell survival. We validated our screening results using RNAi, CRISPRi, and antisense oligonucleotides. We found a lncRNA, termed ASTILCS, that is critical for HCC cell growth and is overexpressed in tumors from HCC patients. We demonstrated that HCC cell death upon ASTILCS knockdown is associated with apoptosis induction and downregulation of a neighboring gene, protein tyrosine kinase 2 (PTK2), a crucial protein for HCC cell survival. Taken together, our study describes a new, non-coding RNA regulator of HCC.

## Introduction

Liver cancer is one of the leading causes of cancer mortality worldwide, accounting for more than 700,000 deaths per year^1^. Hepatocellular carcinoma (HCC) is the most frequent subtype of liver cancer. Despite recent progress in HCC treatment it remains one of the deadliest types of cancer^1,2^. Notably, the incidence of HCC has been increasing in recent decades, making HCC one of the fastest-growing causes of death worldwide^3^. This poor prognosis underlines the need for new effective therapies. Better understanding of the molecular mechanisms regulating HCC progression may yield new potential drug targets.

A meta-analysis of human HCC datasets revealed 935 genes for which RNA expression was significantly dysregulated in HCC samples compared to healthy tissues^4^. Further Gene Ontology analysis of these genes identified several gene networks associated with HCC progression. Among them were upregulation of cell proliferation, downregulation of apoptosis, loss of hepatocyte differentiation, immunosuppression, and activation of proteins acting at an epigenetic level^4^. Comprehensive genomic profiling of patient HCC samples and their comparison with healthy tissues have helped uncover molecular changes promoting the above phenotypic features of HCC^5–9^. Among them, mutations leading to activation of the WNT signaling pathway were most common^5,6^, implicating the WNT pathway as a major driver of hepatocarcinogenesis^7^. Moreover, activation of the WNT pathway is associated with an immunosuppressive microenvironment, another hallmark of HCC progression^8,9^, which emphasizes the role of WNT pathway activity in HCC progression. Other common mutations affected the TERT promoter, TP53, genes regulating cell cycle, PI3K-AKT-mTOR signaling and cell differentiation^5,6^. Notably, up to 50% of clinical HCC samples reported in different studies have a mutation in chromatin modifiers^5,6^, indicating the importance of epigenetic regulation in HCC development.

Besides shedding light on the roles of protein-coding genes, integrative genomic studies have revealed that the majority (>70%) of transcribed sequences in the human genome participate in cell function regulation without producing a protein^10^. Long non-coding RNAs (lncRNAs) are defined as non-coding transcripts longer than 200 nucleotides and represent a large class of non-coding elements, comprising more than 50,000 annotated transcripts to date^11,12^. Pertinently, hundreds of lncRNAs are recurrently deregulated in HCC, suggesting potential roles in hepatocarcinogenesis. Co-expression network analysis determined that these lncRNAs were associated with cell proliferation, metastasis, immune response, and liver metabolism—hallmarks of HCC progression^13,14^. While the pathogenic roles of some of these lncRNAs (e.g. HULC, H19, HOTAIR, HOTTIP, DANCR) have already been described^15^, a plurality of lncRNA transcripts remain largely uncharacterized. Discovery of novel lncRNAs and their intracellular functions promises to expand our knowledge of HCC cellular physiology and may provide the basis for new therapeutic modalities.

Currently, lncRNA functions can hardly be predicted based on their sequence. Instead, subcellular localization, transcript abundance, and functional genomic screens can help to efficiently narrow down possible lncRNA biological roles and molecular functions^16–19^. For instance, lncRNAs located mainly in the nucleus typically function as transcription regulators of local genes (*in cis*) or distant genes (*in trans*)^18^. Cytoplasmic lncRNAs are more likely to regulate protein production, formation of post-translational modifications, and sequestration of miRNAs or RNA-binding proteins^19^. Transcript abundance can provide another hint about lncRNA function. For example, low-abundance transcripts tend to function in *cis* because their low concentration makes diffusion a barrier to activity at long distances from the transcription site. Abundant lncRNAs, on the other hand, can achieve high concentrations at multiple target regions, including those outside of the nucleus and therefore often function in *trans*^16,17^. Finally, pooled functional genetic screens are a powerful tool allowing for parallel perturbation of multiple genes to select for those that are critical for a phenotype or function^20,21^. Recently, genome-wide screens have made it possible to identify lncRNAs involved in a wide variety of cellular functions including cell proliferation, drug resistance, autophagy, tissue homeostasis, and cell differentiation^22–26^.

RNA interference (RNAi) is an effective method for transient silencing of gene expression and therefore is an instrument for loss-of-function genetic screens^20,27^. Previously, it was reported that RNAi-mediated gene silencing is restricted to the cytoplasm, limiting targeting of nuclear transcripts. However, recent studies suggest RNAi presence and activity in the mammalian nucleus as well, although with less efficiency^28–30^. Clustered regularly interspaced short palindromic repeat interference (CRISPRi) is another potent technique for lncRNA silencing^21,23,25^. Given that majority of lncRNA promoters is poorly annotated and lncRNAs often overlap with protein-coding genes (or their promoters/enhancers), application of CRISPRi to regulate a lncRNA overlapping with other transcripts might distort expression of those transcripts, confounding data interpretation^31^. Thus, in our screen we have chosen to perturb lncRNA at the transcript level. We performed an shRNA-based pooled screen to identify lncRNAs essential for the survival of the human HCC cell line HUH7. Based on the lncRNA expression profile of these cells, we designed a lentiviral shRNA library targeting all identified lncRNAs. Using this library, we performed a loss-of-function genetic screen and found that lncRNA ENST00000501440.1 is critical for HUH7 cell growth. We named this lncRNA ASTILCS (Anti**S**ense Transcript Important for Liver Carcinoma Survival). Importantly, in patient data, ASTILCS is significantly overexpressed in HCC compared to normal tissues. Further, using gene expression manipulation techniques, we demonstrate that ASTILCS knockdown results in apoptosis induction and HCC cell death. Finally, we show that ASTILCS knockdown correlates with downregulation of a neighboring gene expressing Protein Tyrosine Kinase 2 (PTK2), the silencing of which results in HCC cell death.

## Materials and methods

### Cell culture

Human HCC HUH7 cell line was a gift from Prof. Jay Horton (UT Southwestern Medical Center). HUH7 and HEK293ft cell lines were grown in Dulbecco’s modified Eagle’s medium with l-glutamine (DMEM, Gibco™) supplemented with 4.5 mg/ml glucose, 50 µg/ml gentamicin sulfate (Sigma), 25 mM HEPES (Gibco™), and 10% heat-inactivated fetal bovine serum (FBS, Gibco™). All cells were cultured at 37 °C, 5% CO_2_. When the cells reached a 70–80% monolayer, they were detached from the flask using 0.25% Trypsin–EDTA (Gibco™) solution and split 1:10. Concentrations for selection agents were determined using killing curve: 2.5 µg/ml puromycin (Sigma), 0.75 mg/ml G-480 (Sigma). The cells were tested for mycoplasma contamination.

### RNA sequencing and data analysis

Samples were prepared using strand-specific Ribo-Zero kit and RNA sequencing was performed by MIT BioMicro Center (https://openwetware.org/wiki/BioMicroCenter:Software#BMC-BCC_Pipeline). Reads were aligned to transcripts derived from the hg19 assembly and the Ensembl version 68 non-coding RNA annotation (non-coding genes) or the full Ensembl 68 annotation (protein-coding genes) using Bowtie version 1.01^32^ and gene expression was summarized using RSEM version 1.2.3^33^.

### Genome-wide screening

Based on HUH7 RNA-sequencing results (Supplemental Fig. 1, Supplemental Table 1), we designed a library of 7873 shRNA vectors allowing to do knockdown of the identified 1618 lncRNAs based on RNAi^34^^,^^35^. The library was developed, synthesized, and packed into lentivirus by the RNAi Consortium at the Broad Institute^36^. The shRNA sequences were assembled into a pLKO.1 lentiviral backbone, containing a puromycin resistance marker to allow for the antibiotic selection of transduced cells. CMV-VSV-G and psPAX2 plasmids were used for lentiviral packaging. The lentiviral library contained four to five shRNAs per target lncRNA and was applied at a low multiplicity of infection (MOI) equal to 0.3. Two days after lentiviral library exposure, infected cells were selected for 4 days on puromycin. To assess effects of shRNAs on cell survival, the selected cells were cultured for 4 more weeks maintaining an shRNA representation of 500 (i.e. each shRNA was expressed on average by 500 cells). The input pooled shRNA plasmid library before virus production was also sequenced and used as a control.

### Next generation sequencing

Samples for Illumina sequencing were prepared following “One Step PCR Preparation of Samples for Illumina Sequencing” protocol from The RNAi Consortium (https://portals.broadinstitute.org/gpp/public/resources/protocols). Briefly, gDNA was isolated using the QIAamp DNA Blood Maxi Kit (Qiagen). Illumina adapter sequences with five-letter barcodes were used to PCR amplify the shRNA-expressing cassette. The samples were multiplexed and sequenced by MIT BioMicroCenter using HiSeq2000 platform. The samples were processed using the BMC/BCC 1.5.2 pipeline (updated on 08/12/2016). Adapter sequence GGAAAGGACGAGGTACC was trimmed from reads using Cutadapt version 1.4.2^37^. Trimmed reads were then aligned target consisting of the 7873 sequence shRNA library with BWA version 0.7.10^38^. Mapped reads were summarized and parsed using SAMtools version 1.3^39^ and custom Perl scripts. The resulting count table was tested for differential representation using DESeq2 version 1.10.1^40^ running under R version 3.2.3. Differential expression data was visualized using Tibco Spotfire Analyst version 7.11.1.

### Molecular cloning

shRNAs from the library (Supplemental Table 2) were annealed and cloned into a pLKO.1_neo plasmid (a gift from Prof. Sheila Stewart; Addgene plasmid # 13425; http://n2t.net/addgene:13425; RRID:Addgene_13425) using a protocol from Wiederschain et al.^41^. Two shRNAs designed to target mCherry were used as controls. Briefly, oligos were resuspended in water to a final concentration of 100 μM. 11.25 μl of each oligo (top and bottom) were mixed with 2.5 μl of 10× annealing buffer (1 M NaCl (Sigma), 100 mM Tris–HCl (Sigma), pH = 7.4) and annealed at 95 °C using a water bath. The pLKO.1_neo plasmid was digested using AgeI and EcoRI restriction enzymes and purified on 1% agarose gel. Next, oligo mixture was diluted 1:400 in 0.5× annealing buffer and ligated with the digested pLKO.1_neo plasmid using T4 DNA ligase (NEB) (3 h at RT). 2 μl of the ligation mixture was used to transform 10 μl of One Shot competent Stbl3 *E. coli* cells (Invitrogen) according to manufacturers’ instructions. Transformed bacteria were plated on LB-agar plates (Teknova) with 100 μg/mL ampicillin (Sigma) and incubated overnight. Individual colonies were picked, inoculated in 3 ml of LB (Sigma) with ampicillin to start miniprep cultures and incubated for 24 h. Miniprep DNA was isolated using QIAGEN Plasmid Mini Kit (Qiagen). shRNA sequences were confirmed by Sanger sequencing (performed by Quintara Biosciences).

sgRNAs (Supplemental Table 3) were designed using the Broad Institute’s GPP sgRNA Designer^42^^,^^43^. Two sgRNAs targeting mouse XIST and blasted against human genome and transcriptome to avoid off-targets were used as controls. Then, the sgRNAs were assembled into a plasmid expressing dead Cas9 (dCas9, Cas9 without endonuclease activity) fused with a transcription inhibitor, the Krüppel-associated box (KRAB) transcriptional repression domain, in a lentiviral backbone containing a puromycin resistance sequence (pLV hU6-sgRNA hUbC-dCas9-KRAB-T2a-Puro, a gift from Prof. Charles Gersbach, Addgene plasmid # 71236; http://n2t.net/addgene:71236; RRID:Addgene_71236)^44^ using Golden Gate assembly reaction as described in ref. ^45^. 2 μl of the ligation mixture were used to transform 10 μl of NEB stable competent *E. coli* (NEB) according to manufacturers’ instructions. Transformed bacteria were plated on LB-agar plates with 100 μg/mL ampicillin and incubated overnight. Individual colonies were picked, inoculated in 3 ml of LB (Sigma) with ampicillin to start miniprep cultures and incubated for 24 h. Miniprep DNA was isolated using QIAGEN Plasmid Mini Kit (Qiagen). sgRNA sequences were confirmed by Sanger sequencing (performed by Quintara Biosciences).

To create a plasmid expressing ASTILCS, it’s full sequence was used to substitute GFP in TRC209 lentiviral plasmid (PGK-Hygro-EF1a-GFP, gift from the Broad GPP)^23^. The cloning and sequence validation were done by Genscript Biotech.

### Lentivirus production and transduction

For transduction, plasmids were packaged into lentivirus through transfection of the plasmids with a packaging plasmid (psPAX2 was a gift from Prof. Didier Trono (Addgene plasmid # 12260; http://n2t.net/addgene:12260; RRID:Addgene_12260) and an envelope plasmid CMV-VSV-G was a gift from Prof. Weinberg (Addgene plasmid # 8454; http://n2t.net/addgene:8454; RRID:Addgene_8454)^46^ using TransIT-LT1 Transfection Reagent (Mirus Bio). ~300,000 HEK293ft cells were plated per well into a sx-well plate and incubated overnight. 0.4 μg PAX2, 0.15 VSV-G and 3.3 μg plasmid of interest were added to 600 μl Opti-MEM (Gibco™) and mixed with an equal volume of Opti-MEM containing 4 μl of TransIT-LT1. The mixture was incubated at RT for 20 min and transferred to the well. The volume was brought to 600 ml per well with the culture media and incubated overnight. On the following day, the media was changed. Media with lentiviral particles was collected after 48 h and snap-frozen in liquid nitrogen. All shRNA/sgRNA plasmids were produced in parallel.

### Arrayed screening

Equal numbers of HUH7 cells (~5000) were plated in a 96-well plate and transduced with 5 μl of shRNAs or 2 μl of sgRNAs packed into lentiviral particles, so that each well received only one type of shRNA/sgRNA. A plasmid expressing green fluorescent protein (GFP) (pLJM1-EGFP was a gift from Prof. David Sabatini (Addgene plasmid # 19319; http://n2t.net/addgene:19319; RRID:Addgene_19319))^47^, but not caring antibiotic resistance marker was also packed into lentiviral particles and used as a positive control for transduction and antibiotic selection. After an overnight incubation the cell media was changed. Two days after the lentiviral transduction, a selection reagent (G-480 or puromycin, respectively) was added to the culture media to select for cells containing the shRNA/sgRNA expressing plasmids. Once the selection was completed (i.e. all non-infected GFP-treated cells were dead), cell survival was measured using Cell Titer assay.

### Cell survival assay

HUH7 cell survival was analyzed using the CellTiter-Glo^®^ Luminescent Cell Viability Assay (Promega) according to the manufacturer’s protocol. Luminescence was measured with the microplate reader Tecan Infinite^®^ 200 PRO.

### Cell proliferation assay

Cells were plated at low density in 96-well plates (2000 cells/well). Cell number analysis using cell titer assay was performed at 1, 2, 3, and 5 days afterwards. For growth curves analysis, doubling time was calculated from the exponential portion of the cell growth curve using the following equation: *T*_d_ = 0.693t/ln(*N*_*t*_/*N*_0_), where *t* is the time (in days), *N*_0_ is initial cell number, *N*_t_ is cell number on day *t*.

### Gene expression analysis

For single tube reactions (Fig. 3G) RNA was isolated using Omega Bio-tek’s E.Z.N.A.^®^ Total RNA Kit I isolation kit according to manufacturers’ instructions. Separation and purification of cytoplasmic and nuclear RNA (Fig. 3E) were done using Cytoplasmic and Nuclear RNA Purification Kit (Norgen Biotek Corp.) also following manufacturers’ instructions. Reverse transcription reaction was performed using Applied Biosystems™ High-Capacity RNA-to-cDNA™ Kit and 1 μg of RNA. RNA levels were assessed by qPCR using Power SYBR™ Green PCR Master Mix (Applied Biosystems™). For high-throughput experiments (all other RNA expression figures) RNA isolation, reverse transcription reaction, and qPCR was performed using Power SYBR™ Green Cells-to-CT™ Kit (Ambion) according to manufacturers’ instructions. TaqMan Fast Advanced Master Mix (Applied Biosystems™) was used with TaqMan primers (Hs01060665_g1 for ACTB and Hs01377184_m1 for ASTILCS, both from ThermoFisher) (Fig. 2B). β-actin mRNA was used as a housekeeping control. The RNA levels were first normalized to the level of β-actin and then to an average value of the control group. All SYBR Green primers are listed in Supplemental Table 7.

### LNA transfection

LNA gapmers targeting ASTILCS, SLC45A, PTK2, and PTP4A3 genes were custom-designed using Qiagen’s Antisense LNA GapmeR designer (Supplemental Table 4), and non-targeting LNA gapmer (Negative Control A (NCA)) was included as a control. LNA gapmers were resuspended in water to a final concentration of 50 μM. 10,000 HUH7 cells were plated per well in a 96-well plate and incubated overnight. LNA gapmers were formulated with Lipofectamine 2000 (Invitrogen) in Opti-MEM (Gibco™) according to the manufacturer. Each well was treated with 50 μl Opti-MEM containing 20 pmol LNA gapmer formulated and 0.25 μl Lipofectamine 2000. Cell survival and gene expression were measured 24 h after transfection.

### Apoptosis analysis

It was performed using In Situ Cell Death Detection Kit, TMR red (Roche) according to manufacturers’ instructions. Briefly, cells were collected using 0.25% Trypsin–EDTA solution (Gibco™), fixed with 4% paraformaldehyde (Electron Microscopy Sciences) in PBS (Gibco™) at RT for 30 min and permeabilized with 0.1% Triton X-100 (Sigma) in 0.1% sodium citrate (Sigma) for 2 min on ice. Next, TUNEL reaction mixture was added to the cells and incubated at 37 °C for 60 min. TMB-positive cells were detected and counted using BD FACSCelesta Flow Cytometer, at least 10,000 cells were analyzed per sample.

### Cell cycle analysis

It was performed using Click-iT EdU Alexa Fluor 647 Flow Cytometry Assay Kit (Invitrogen) and FxCycle Violet Stain (Invitrogen) according to manufacturers’ instructions. Briefly, cells were labeled with 10 μM EdU for 45 min. Then the cells were harvested using a 0.25% Trypsin–EDTA solution (Gibco™), fixed with Click-iT fixative at RT for 15 min protected from light and permeabilized with Click-iT saponin-based permeabilization and wash reagent. Next, click-iT^®^ reaction cocktail was added to the cells and incubated for 30 min at RT protected from light. Finally, 1 μL of FxCycle™ Violet stain was added to each sample and incubated for 30 min at RT protected from light. Fluorescence was analyzed using BD FACSCelesta Flow Cytometer, at least 10,000 cells were analyzed per sample.

### Statistical analysis

Statistical significance was calculated using GraphPad Prism 8.2 package. The sample size was chosen based on preliminary results and similar publications. ROUT test (*Q* = 1%) was used to identify and remove outliers. D’Agostino–Pearson omnibus normality test was used to establish whether or not the population is distributed normally. Unpaired Mann–Whitney test was used to calculate the difference between two different populations which are not normally distributed. One-way analysis of variance (ANOVA) followed by Dunnett’s post hoc test was used for multiple comparisons analysis of normally distributed populations with equal variances (i.e. equal standard deviations (SD)). Brown–Forsythe and Welch ANOVA tests followed by Dunnett’s T3 multiple comparisons analysis were used for normally distributed populations with different SDs. Kruskal–Wallis test followed by Dunn’s multiple comparisons analysis was used for populations which are not normally distributed.

## Results

### Pooled RNAi-based screen identifies lncRNAs potentially essential for HCC cell survival

**Fig. 1:**
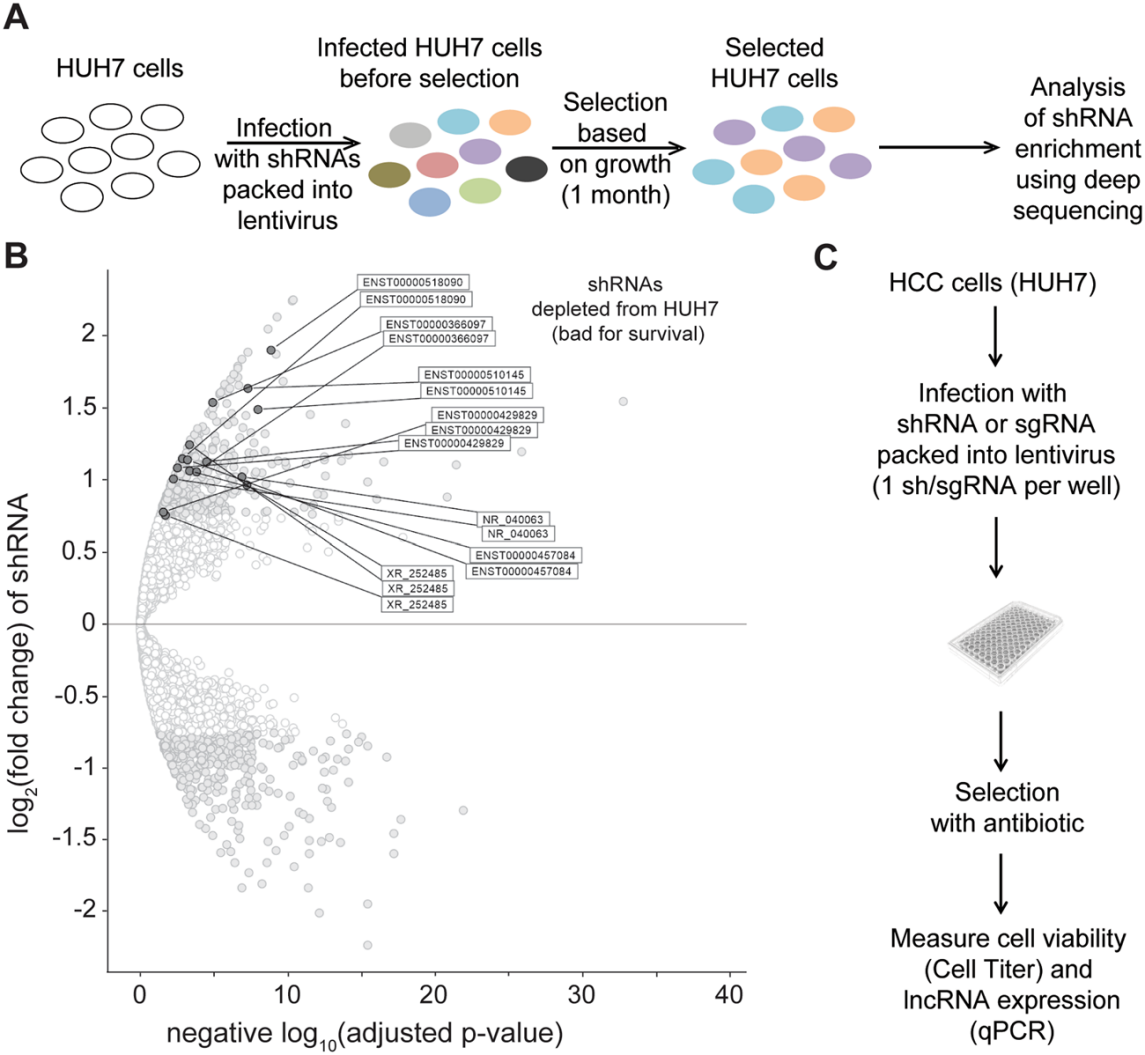
Experimental design and selection strategy for the identification of lncRNAs essential for HUH7 HCC cell survival. **A** Schematic workflow of the survival-based pooled shRNA library screen in HUH7 cells. shRNAs were designed to target lncRNAs identified in the cell line. **B** Volcano plot of the differentially expressed shRNAs in the final population of HUH7 cells. The *x*-axis indicates the adjusted *p*-values plotted in −log_10_. The *y*-axis indicates the log_2_(fold change) in shRNA expression, which was defined as the ratio of normalized shRNA expression in the input library over the final HUH7 population. Light gray dots represent shRNAs with log_2_(fold change) ≥ 0.75 and adjusted *p*-value ≤ 0.05. Dark gray dots represent shRNAs of lncRNAs for which at least two shRNAs have log_2_(fold change) ≥ 1 and adjusted *p*-value ≤ 0.05 or lncRNAs for which at least three shRNAs have log_2_(fold change) ≥ 0.75 and adjusted *p*-value ≤ 0.05. **C** Schematic workflow of arrayed shRNA and sgRNA screens used for validation of lncRNAs identified in **B**.

To design the shRNA library, we performed transcriptome analysis in HUH7 HCC cell line and identified 1618 non-coding RNA transcripts longer than 200 base pairs and expressed at a level higher than 5 FPKM (Supplemental Fig. 1, Supplemental Table 1). Next, we constructed a library of 7873 shRNA vectors to knockdown the identified lncRNAs based on RNAi and applied on HUH7 cells (Fig. 1A). Each lncRNA was targeted by 4–5 shRNAs to account for shRNA off-target effects. To identify lncRNAs important for HUH7 cell survival, shRNAs present in the final population were compared to shRNA representation in the input library. A lncRNA was considered a candidate when at least two of its corresponding shRNAs were underrepresented in the final population with log_2_(fold change compared to control) ≥ 1 or by at least three shRNAs with log_2_(fold change compared to control) ≥ 0.75 (Fig. 1B). With these constraints, we identified seven lncRNA candidates for further validation (ENST00000429829, ENST00000510145, ENST00000457084, ENST00000501440.1, ENST00000366097.2, ENST00000518090, and ENST00000421703.5). To the best of our knowledge only ENST00000429829 and ENST00000510145 were previously characterized^48–54^.

Both of these lncRNAs have been identified in the context of cancer. Although their mechanisms are the focus of active discussion, their presence among our screen hits supports the likelihood that the rest of the transcripts are also involved in HCC cell survival and biology. ENST00000429829 is one of the multiple transcripts of gene ENSG00000229807, also known as XIST. In addition to its established role as the master regulator of X chromosome inactivation^55^, XIST has been reported to participate in progression of a variety of cancers, including HCC^48–52^. However, the results of these studies are controversial^48–52^. ENST00000510145 is one of nine transcripts of gene ENSG00000250682, also known as LINC00491 or BC008363. This gene was found to be upregulated in a TCGA colon adenocarcinoma dataset and was associated with lower patient survival, implying ENSG00000250682 importance for colorectal cancer progression^53^. Conversely, in pancreatic ductal adenocarcinoma patients LINC00491 expression was significantly lower compared to the control group and was associated with better survival rates^54^.

### Validation of the screen results identifies lncRNA ASTILCS as a new regulator of HCC cell survival

**Fig. 2:**
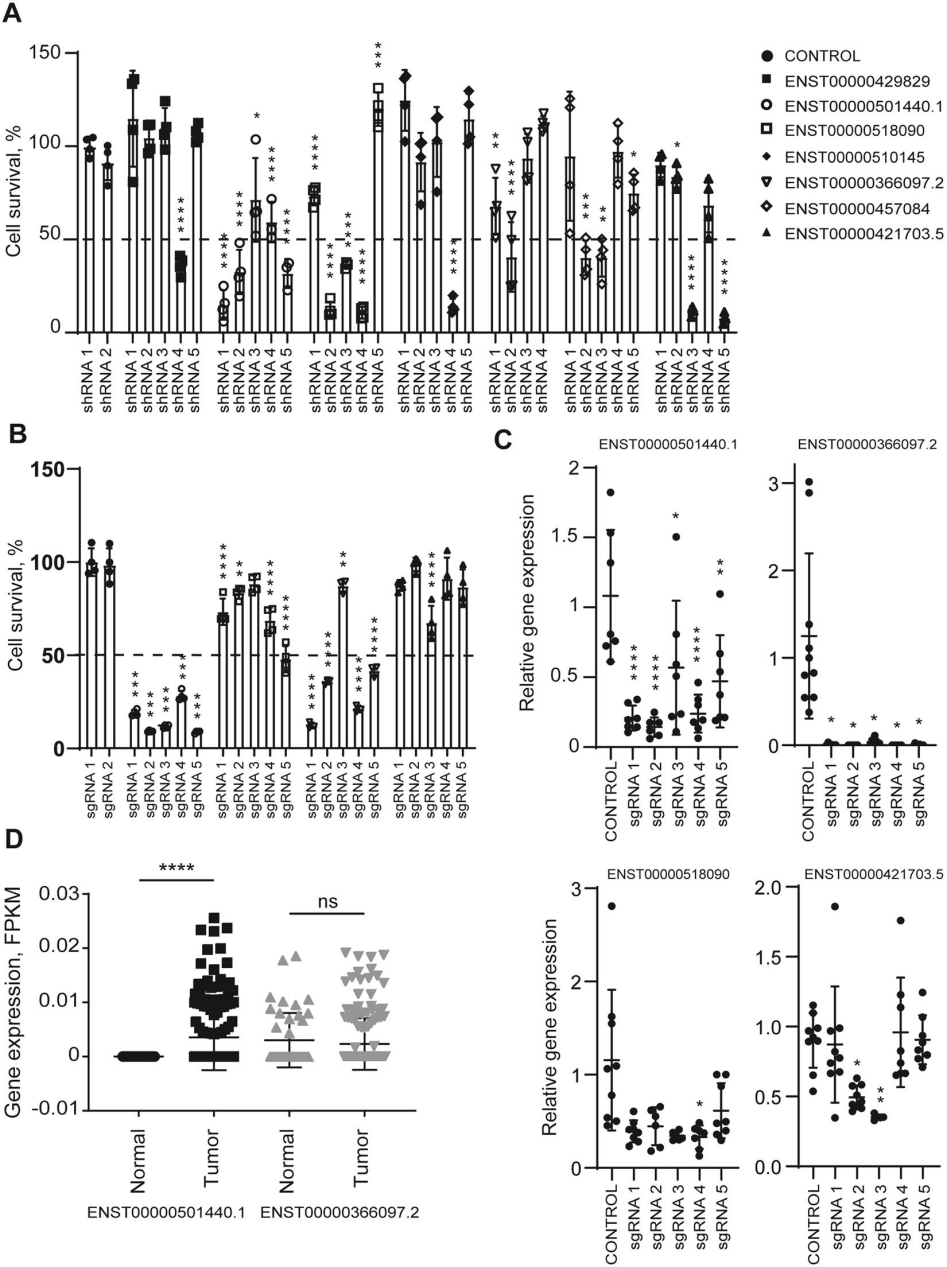
Validation of the screen results identifies lncRNA ASTILCS a new regulator of HCC cell survival. **A** HUH7 cell survival upon shRNA-mediated knockdown of candidate lncRNAs (compared to control shRNA1), *n* ≥ 3. **B** HUH7 cell survival upon CRISPRi-mediated knockdown of lncRNA candidates from **A** compared to control sgRNA1, *n* ≥ 3. **C** LncRNA expression in HUH7 cells transduced with sgRNA-dCas9-KRAB targeting one of the lncRNA candidates, *n* ≥ 4. **D** ENST00000501440.1 (ASTILCS) and ENST00000366097.2 expression in HCC vs. adjacent tissue in patient samples (TCGA-LIHC-rnaexp downloaded from TANRIC), *n* ≥ 45. All values are mean ± SD, **** - *p* < 0.0001; *** - *p* < 0.001; ** - *p* < 0.01; * - *p* < 0.05.

To validate the screening results, we individually expressed the five library shRNAs for each of the seven candidate lncRNAs (Supplemental Table 2) and repeated the screen in an arrayed format (Fig. 1C). Those lncRNAs for which at least two corresponding shRNAs reduced cell survival by more than 50% compared to the control shRNAs were selected for further analysis; these were ENST00000501440.1, ENST00000366097.2, ENST00000518090, and ENST00000421703.5 (Fig. 2A).

RNAi-based gene silencing is associated with a few pitfalls, particularly off-target activity and variability in knockdown efficiency^56^^,^^57^. We therefore further validated the candidate lncRNAs using an arrayed screen based on CRISPRi (Fig. 1C). To do so, we designed five sgRNAs (Supplemental Table 3) to allow targeting of each candidate lncRNA by CRISPRi (Fig. 2B, C). Among the four studied lncRNAs, CRISPRi-mediated knockdown of only ENST00000501440.1 and ENST00000366097.2 resulted in substantially decreased survival for HUH7 HCC cells (Fig. 2B, C). Specifically, 5/5 sgRNAs targeting lncRNA ENST00000501440.1 decreased HCC cell survival by more than 70% and 4/5 sgRNAs targeting lncRNA ENST00000366097.2 resulted in more than 50% HUH7 cell death. In contrast, knockdown of ENST00000518090 was not associated with a notable decrease in HCC cell survival and only 2/5 sgRNAs designed to target ENST00000421703.5 induced partial lncRNA knockdown with mild effects on HCC cell survival. Discrepancy between the survival data obtained with RNAi and CRISPRi for ENST00000518090 and ENST00000421703 (Fig. 2A versus Fig. 2B) can be due to the differences in the mechanisms of the knockdown methods. In fact, multiple comparison studies have been published demonstrating that these approaches are complementary and not always identical, especially in case of lncRNAs^21^^,^^58^. Based on these results we concluded that ENST00000501440.1 and ENST00000366097.2 gene expression is critical for HCC cell survival. ENST00000501440.1 is the only transcript of ENSG00000244998 gene. It is a 1380 bp long antisense transcript comprised of two exons. ENST00000366097.2 is one of two transcripts of ENSG00000203266 gene. It is a 770 bp long intergenic lncRNA consisting of three exons. Both transcripts (ENST00000501440.1 and ENST00000366097.2) are predicted to have low coding potential and are not conserved in chimpanzee or mouse^59^. Thus, we identified two novel lncRNA genes which expression is potentially important for HCC cell survival.

To determine whether these two lncRNAs are HCC specific or are present in healthy liver tissues, we examined ENST00000501440.1 and ENST00000366097.2 gene expression in tissue samples from patients with HCC using a dataset from The Cancer Genome Atlas (TCGA-LIHC-rnaexp, downloaded from The Atlas of NcRNA in Cancer (TANRIC))^60^. We found that ENST00000501440.1 expression was significantly higher in liver cancer samples compared to the adjacent tissue (Fig. 2D; *p* < 0.0001). These data suggest that only ENST00000501440.1 expression is critical for the survival of tumor cells. Yet, lncRNA expression was not associated with patient survival (Supplemental Fig. 2; long-rank *p*-value = 0.5)^60^. Because only ENST00000501440.1 expression is differentially expressed in cancer cells, we selected it for further analysis. Through the rest of the publication, we refer to this lncRNA by the name of ASTILCS (AntiSense Transcript Important for Liver Carcinoma Survival).

**Fig. 3:**
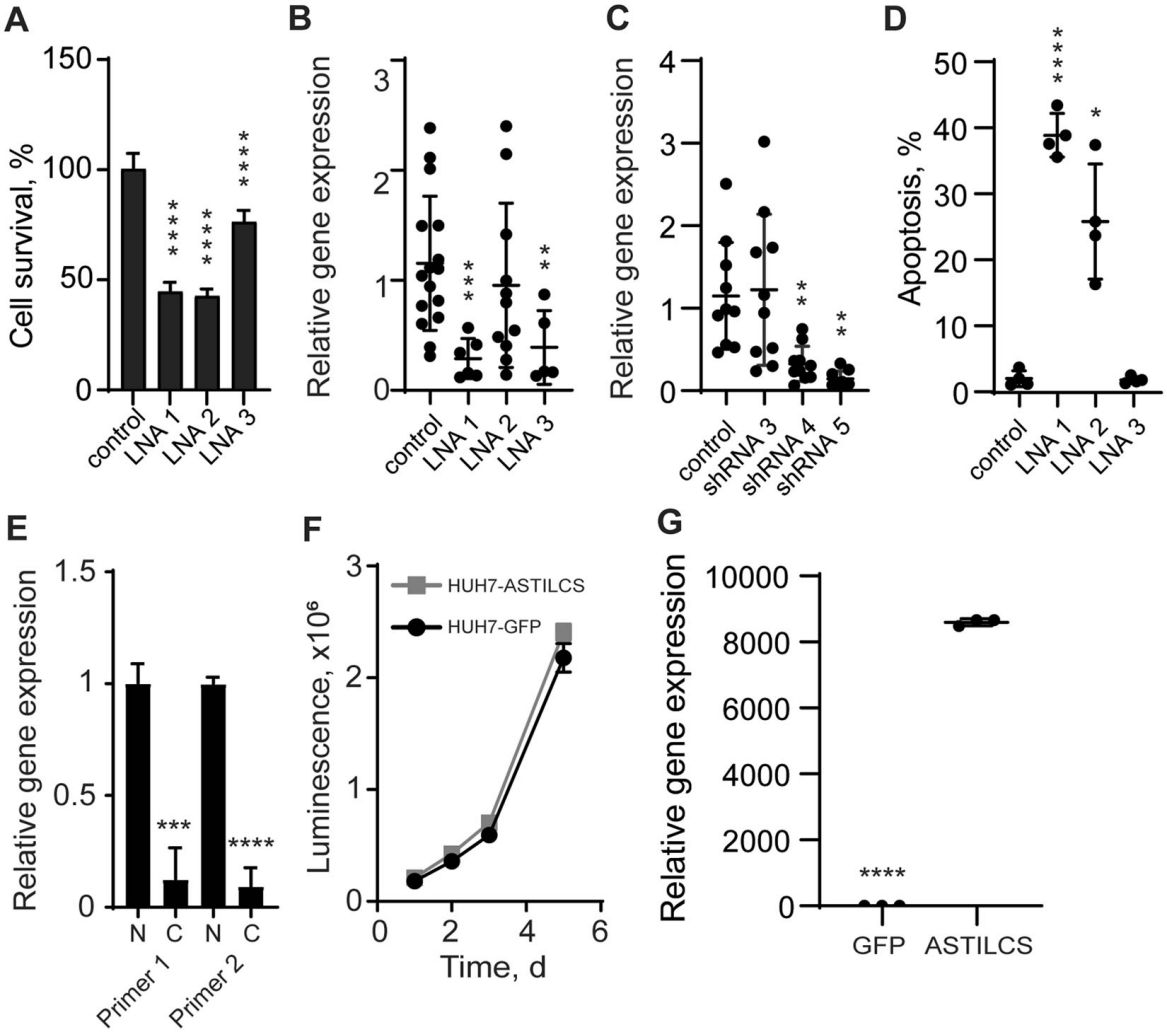
ASTILCS expression is essential for liver carcinoma cell survival. **A** HUH7 cell survival 48 h after transfection with LNA gapmers targeting ASTILCS, *n* ≥ 6. **B** ASTILCS expression in HUH7 cells transfected with LNA gapmers targeting ASTILCS, *n* ≥ 5. **C** ASTICLS expression in HUH7 cells transduced with shRNAs targeting ASTILCS, *n* ≥ 5. **D** Apoptosis in HUH7 cells treated with LNA gapmers targeting ASTILCS, *n* = 3**. E** ASTILCS expression in HUH7 cell nucleus and cytoplasm, *n* ≥ 8. **F** Growth curve for HUH7 cells transfected with GFP- or ASTILCS-expressing plasmids. **G** ASTILCS expression in HUH7 cells transduced with GFP- or ASTILCS-TRC209, *n* = 3. All values are mean ± SD, **** - *p* < 0.0001; *** - *p* < 0.001; ** - *p* < 0.01; * - *p* < 0.05; ns. - *p* > 0.05.

An insight into ASTILCS locus revealed that ASTILCS is an antisense sequence to the protein-coding gene Protein Tyrosine Phosphatase Type IVA 3 (PTP4A3) (Supplemental Fig. 3). PTP4A3 is important for cell proliferation; its knockdown decreases cell survival in multiple types of cells^61–65^. Because sgRNAs targeting ASTILCS bind PTP4A3 between 512 and 611 bp away from the transcription start site, there is a possibility that the sgRNA–dCas9–KRAB complex hinders PTP4A3 mRNA expression, resulting in HCC cell death independently of ASTILCS. Indeed, gene expression analysis of the sgRNA-treated cells revealed deep knockdown of PTP4A3 (Supplemental Fig. 4). To add orthogonal evidence of ASTILCS prosurvival effects on HCC cells, we knocked down its expression by transient transfection of antisense oligonucleotides containing locked nucleic acid modifications (LNA) (Supplemental Table 4). LNA gapmers bind with high affinity to complementary RNA sequences forming DNA•RNA hybrids, which are recognized and cleaved by RNAse H1, resulting in efficient gene knockdown^66^. We observed reduction in HUH7 HCC cell survival upon treatment with the LNA gapmers (Fig. 3A), which was associated with ASTILCS knockdown (Fig. 3B). We noticed that, despite a decrease in cell survival in LNA gapmer 2-treated samples, ASTILCS RNA levels in these samples were not significantly affected. These findings may be explained by previous reports demonstrating that antisense oligonucleotide hybridization with RNA can affect its function without inducing degradation^67^^,^^68^. Thus, LNA gapmer 2 binding to ASTILCS might perturb its function via steric blocking of lncRNA secondary structure formation or interaction with molecules important for the lncRNA signaling^69^^,^^70^. To further corroborate whether ASTILCS expression is critical for HCC cell survival, we measured its expression in HUH7 HCC cells transfected with the three most efficient shRNAs from the library (Supplemental Fig. 5) and observed dosage-dependent decrease in HCC cell survival (Figs. 3C and 2A). These findings substantiate that ASTILCS regulates HCC cell survival and its specific knockdown leads to HCC cell death independently of its reciprocal sense coding gene, PTP4A3.

### LncRNA ASTILCS knockdown in HCC cells results in apoptosis induction

To understand the molecular mechanism of ASTILCS effects on HUH7 cell survival, we studied whether ASTILCS knockdown affects HUH7 cell cycle distribution and apoptosis. To that end, we performed an EdU-FxCycle Violet assay to determine ASTILCS effects on cell cycle. We did not observe any changes in cell cycle distribution which would correlate with ASTILCS knockdown efficacy in samples treated with shRNAs or with LNA gapmers compared to untreated control (Supplemental Fig. 6). Thus, we concluded that ASTILCS knockdown does not affect HUH7 cell proliferation in 24–48 h after transfection. Even if ASTILCS influence the transcription of cell cycle regulators, the effect can be postponed until a protein regulator will be degraded.

Next, we performed a TUNEL assay to assess ASTILCS knockdown effects on HUH7 cell apoptosis. We found that transformation with shRNA expressing plasmids or treatment with LNA gapmers led to a dose-dependent increase in the number of apoptotic cells (Fig. 3D and Supplemental Fig. 7). Differences in the apoptotic cell number between shRNA and LNA gapmer-treated samples were likely due to experimental constraints in the knockdown techniques. Apoptosis levels in LNA gapmer-treated samples were measured 24 h after the treatment, while in shRNA-treated samples apoptosis could only be measured 4 days after transduction, providing time for compensation mechanisms to occur. Moreover, cell media in shRNA-treated samples had to be changed to remove the lentiviral particles and add selective agent, which could also result in partial removal of poorly attached apoptotic cells. From our findings we conclude that ASTILCS knockdown results in the induction of apoptosis and a subsequent decrease in HUH7 cell survival.

### ASTILCS is a nuclear antisense transcript which functions in *cis*

As subcellular localization can hint towards the molecular mechanism of a lncRNA, we measured ASTILCS transcript levels in nuclear and cytoplasmic extracts and found ASTILCS RNA to be strongly enriched in the nucleus (Fig. 3E). These results are in line with the relatively low expression level of ASTILCS transcript in HUH7 cells (~23.5 FPKM, Supplemental Table 5), a common feature of nuclear transcripts. Further, to classify the mechanism by which ASTILCS knockdown decreases HCC cell survival, we determined whether ASTILCS functions in *cis* or *trans*. To do so, we overexpressed cDNA encoding ASTILCS from a randomly integrated lentivirus and assessed cell proliferation as the population doubling time (*T*_d_). We found that the *T*_d_ of cells overexpressing ASTILCS (1.13 ± 0.07 days) was similar to the *T*_d_ of control cells expressing GFP from the same vector (1.13 ± 0.03 days, *p* = 0.17) (Fig. 3F,G and Supplemental Table 6). Because we did not observe any gain in survival for cells overexpressing ASTILCS, we concluded that ASTILCS is not likely to act in *trans* and that its effects on HCC cell survival are probably associated with *cis* functions.

### ASTILCS silencing is associated with downregulation of neighboring gene PTK2 essential for HCC cell survival

**Fig. 4:**
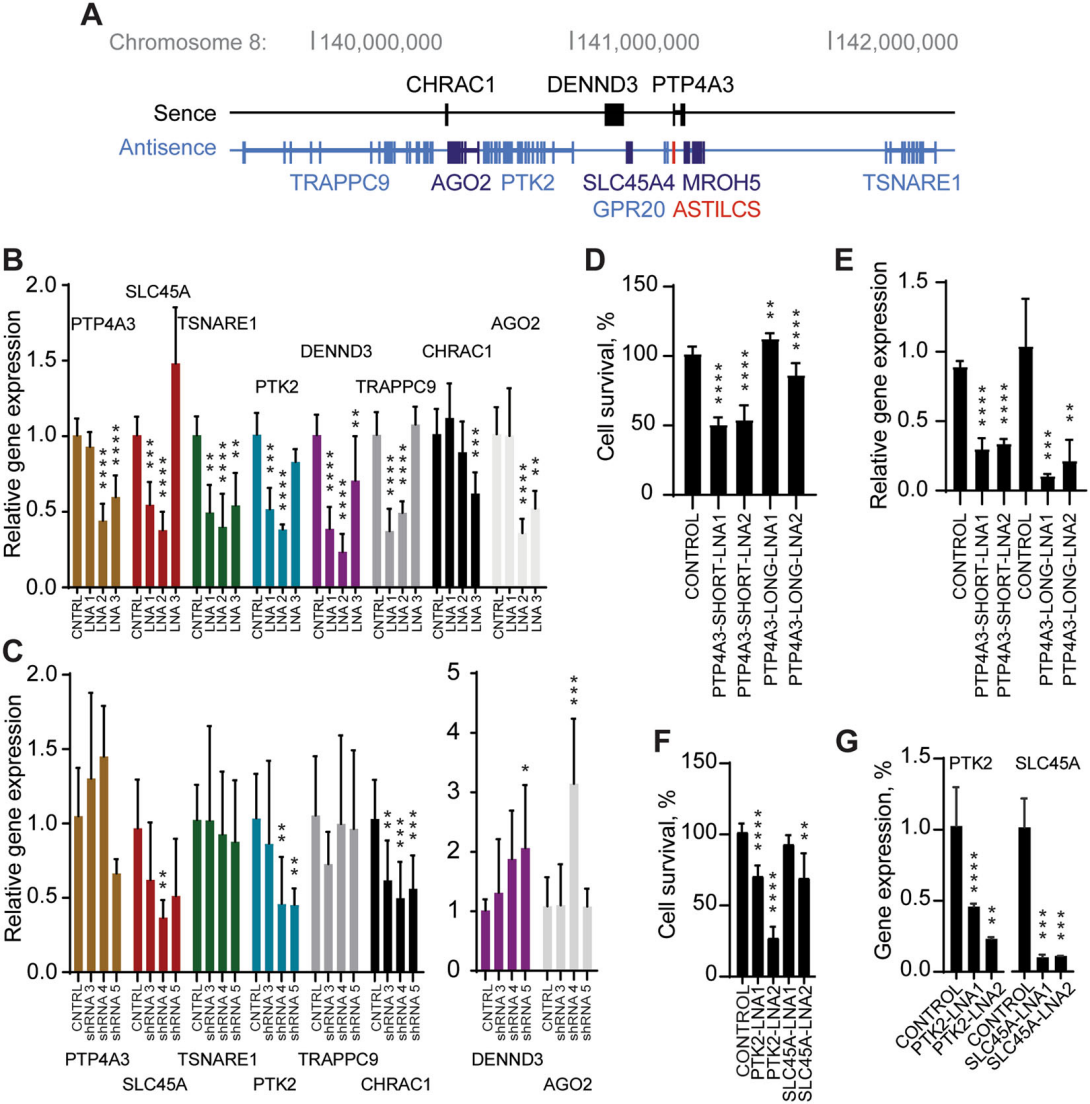
Knockdown of ASTILCS results in dose-dependent downregulation of neighboring genes. **A** Genomic locus of ASTILCS. RNA expression of ASTILCS neighboring genes in HUH7 cells upon LNA gapmer-mediated (*n* ≥ 8) (**B**) or shRNA-mediated (*n* ≥ 5) (**C**) knockdown of ASTILCS. Cell survival (*n* ≥ 8) (**D**) and gene expression (*n* ≥ 5) (**E**) upon LNA-mediated silencing of PTP4A3 isoforms. Cell survival (*n* ≥ 9) (**F**) and gene expression (*n* ≥ 5) (**G**) upon LNA gapmer-mediated silencing of ASTILCS neighboring genes PTK2 or SCL45A, *n* ≥ 9. All values are mean ± SD, **** - *p* < 0.0001; *** - *p* < 0.001; ** - *p* < 0.01; * - *p* < 0.05.

The effects of low abundance nuclear *cis*-acting lncRNAs occur typically in the loci from which they are transcribed. Those effects can be mediated by: (1) the lncRNA transcripts themselves or in complexes with proteins like PRC2; (2) the act of lncRNA transcription; or (3) the regulatory DNA elements within the lncRNA locus^70^^,^^71^. To determine whether the investigated phenotype might result from ASTILCS transcript effects on local gene expression, we examined the impact of ASTILCS knockdown on the expression of all genes within 1 Mb of the target site (Fig. 4A). Analysis of the HUH7 HCC cell transcriptome revealed that G protein-coupled receptor 20 (GPR20) and Maestro Heat Like Repeat Family Member 5 (MROH5) are not expressed in HUH7 cells (Supplemental Table 6), so they were removed from consideration. We found that LNA gapmer-induced ASTILCS knockdown led to a change in expression of all studied genes in the locus (Fig. 4B). Only downregulation of Solute Carrier Family 45 Member 4 (SLC45A4), Protein Tyrosine Kinase 2 (PTK2), DENN Domain Containing 3 (DENND3), and Trafficking Protein Particle Complex 9 (TRAPPC9) was associated with a dose-dependent decrease in both ASTILCS expression and HCC cell survival (Fig. 4B, see also Fig. 3A, B). In contrast, shRNA-mediated knockdown of ASTILCS was associated with gene expression downregulation of SLC45A, PTK2, and Chromatin accessibility complex protein 1 (CHRAC1) (Fig. 4C, see also Figs. 2A, 3C). Expression of genes that was inconsistent across ASTILCS knockdown approaches was considered to result from indirect or off-target effects. Because only SLC45A and PTK2 mRNA expression was affected in the same manner by both shRNAs and LNA gapmers, we inferred that ASTILCS knockdown potentially induces HCC cell death by downregulating expression of one or both of these genes.

LncRNAs transcribed in opposite (antisense) direction to protein coding genes are often found to regulate activity of their sense pair in different manners^72^^,^^73^. Surprisingly, even though protein-coding gene PTP4A3 is located antisense to ASTILCS, we did not observe an apparent effect of ASTILCS knockdown on PTP4A3 mRNA expression (Fig. 4B, C). This fact indicates that the ASTILCS transcript itself does not affect the expression of PTP4A3 gene. Next, we studied whether PTP4A3 knockdown can affect HUH7 HCC cell survival. PTP4A3 produces six transcripts (T1–6), three longer (T3–5) than others (T1,2,6) (Supplemental Fig. 8); the sequence of only the long transcripts overlaps with ASTILCS. We designed LNA gapmers targeting long isoforms of PTP4A3 (T3–5)—PTP-LONG-LNA and LNA gapmers targeting two (T1,2) out of three short isoforms of PTP4A3. We could not design an LNA gapmer targeting only isoform T6 because it completely overlaps with the long isoforms. Interestingly, we found that knockdown of only the short PTP4A3 isoforms led to a dose-dependent decrease in HCC cell survival (Fig. 4D, E). Intrigued by the results, we also analyzed whether knockdown of the long PTP4A3 isoforms can affect the gene expression of the short isoforms. With this mechanism in mind, we measured the expression of the short isoforms in HUH7 HCC cells treated with LNA gapmers targeting long isoforms and observed no difference in the transcript expression (Supplemental Fig. 9). Because ASTILCS overlaps only with the long PTP4A3 isoforms and their knockdown does not affect the expression of their short, survival modulating counterparts, we conclude that ASTILCS silencing does not lead to a decrease in cell survival via downregulation of PTP4A3 transcripts.

Finally, we studied whether RNA knockdown of SLC45A and PTK2 itself can decrease HCC cell survival. PTK2 has been previously shown to affect HCC cell survival, with PTK2 silencing in HepG2 and HUH6 HCC cells lines reducing cell growth and inducing apoptosis^74^. Meanwhile, SLC45A4 has not been reported to affect cell survival. We treated HUH7 HCC cells with LNA gapmers targeting SLC45A or PTK2 and measured cell survival. We observed that only knockdown of PTK2 gene was associated with a decrease in HCC cell survival (Fig. 4F, G). Based on our results we conclude that ASTILCS knockdown might decrease HUH7 cell survival and induce apoptosis via downregulation of PTK2.

## Discussion

Despite recent progress in HCC management, it remains the second deadliest cancer type with a 5-year relative patient survival rate of only 18%^1,2^. A better understanding of HCC biology informs the development of more efficient treatment strategies. An increasing number of studies suggests a vital role for lncRNAs in HCC progression^75–77^. However, their functions in HCC biology remain largely unexplored. To address that problem, in our study, we performed an shRNA-based pooled functional genetic screen to find lncRNAs that play crucial roles in HCC cell progression. Applying stringent filtering criteria and three-step validation we identified lncRNA ASTILCS (ENST00000501440.1) to be important for survival of HCC cells. To the best of our knowledge, we provide the first characterization of the lncRNA ASTILCS. Following a framework suggested by Joung et al. in ref. ^23^ we determined that ASTILCS is a nuclear lncRNAs with a local regulatory mechanism. Using gene manipulation techniques, we demonstrated that ASTILCS loss-of-function results in apoptosis and downregulation of the neighboring gene PTK2, suggesting a possible mechanism of ASTILCS antisurvival effect.

PTK2, also known as Focal Adhesion Kinase, is a protein tyrosine kinase that plays an essential role in formation of cell–matrix junctions (focal adhesions), regulation of cell migration, and viability in a variety of cell types^78^. PTK2 recruitment to focal adhesions triggers PTK2 phosphorylation, creating a docking site for SH2 domain-containing proteins (Grb2, Shc, etc.), thus, linking PTK2 to the activation of the pro-proliferative and anti-apoptotic RAS pathway^78^. Besides that, under certain cellular stress conditions, PTK2 can be recruited to the nucleus to facilitate Mdm2-dependent ubiquitination of tumor suppressor protein p53 and downregulate apoptosis^79^. Multiple studies report on the importance of PTK2 for cancer progression^80^^,^^81^. To date a few PTK2 inhibitors have been studied in clinical trials, however, the best observed response was stable disease^82–84^. Understanding of mechanisms of PTK2 regulation might help to develop more effective PTK2-targeting therapies. Recently, two independent scientific groups simultaneously demonstrated that PTK2 is essential for HCC formation and growth in vivo because of its role in activation of the WNT/β-catenin signaling. PTK2 overexpression stimulated β-actin accumulation in the cell nucleus, thereby enhancing transcription of β-actin target genes and promoting hepatocarcinogenesis. PTK2 silencing, on the other hand, led to increase in apoptosis and a decrease in tumor growth^85^^,^^86^. Thus, downregulation of PTK2 mRNA expression by ASTICLS knockdown can be an important factor mediating the mechanism of ASTILCS’ proapoptotic effect in HCC cells.

The molecular mechanisms of ASTILCS increasing PTK2 mRNA expression will require further studies. Epigenetic regulation might be one of the possible mechanisms. PTK2 is overexpressed in 30–60% of HCC patients and is associated with a higher metastasis rate and reduced survival. Meanwhile, PTK2 expression in healthy liver tissues is negligible, which underlines the importance of PTK2 expression for HCC progression^87^^,^^88^. In this study, we found that ASTILCS levels were also significantly increased in HCC samples compared to normal tissues. Interestingly, DNA sequence analysis in HCC patient samples revealed that PTK2 is amplified in 19–26% of cases, but mutated only in 2.5%^5^^,^^87^. Therefore, there should be additional epigenetic mechanisms activating PTK2 expression. Examination of the PTK2 promoter demonstrated that the total methylation level of its CpG islands negatively correlated with PTK2 gene expression. Thus, promoter demethylation might be a mechanism of PTK2 overexpression. Indeed, treatment of HCC cells with a demethylation agent has shown to increase PTK2 mRNA and protein levels^85^. Some lncRNAs are known to affect DNA methylation via direct interaction with DNA methyltransferases (DNMTs) or via indirect recruitment of DNMTs through an intermediate protein^89^. Hence, the aforementioned evidence creates a possibility that ASTILCS can increase PTK2 mRNA expression via regulation of its promoter methylation. More work is needed to explore molecular mechanism underling this process.

In addition to studies how ASTILCS effects on PTK2 RNA expression, we explored ASTILCS relationship with other neighboring genes. One of them, SLC45A4, is a proton-associated sucrose transporter, for which there are no reports of direct association with cancer or cell survival (PubMed search on 08-Jan-2021). In this study, we demonstrate for the first time, that ASTILCS knockdown leads to SLC45A4 gene silencing and that SLC45A4 silencing does not affect cell survival in HCC cells. Surprisingly, we did not observe a direct linkage between knockdown of antisense lncRNA ASTILCS and expression of its sense protein-coding pair, PTP4A3 gene. Thus, we inferred that the decrease in HCC cell survival upon ASTILCS knockdown is not likely mediated by changes in PTP4A3 mRNA expression. PTP4A3, also known as Phosphatase of Regenerating Liver 3 (PRL-3), is a protein-tyrosine phosphatase implicated in both cell proliferation and invasion in several types of cancer, including HCC^64,65^. Despite the importance of PTP4A3 for HCC cell survival, it seems the pro-survival effect of PTP4A3 is not regulated by ASTICLS RNA expression. Yet, this does not exclude the existence of other regulatory mechanisms between ASTILCS and PTP4A3, nor their importance in still undiscovered cell functions. Interestingly, the functional analysis of PTP4A3 transcripts presented here suggests that different transcripts affect cell survival in different ways in HCC cells. For the first time we report that only knockdown of short PTP4A3 transcripts (T1 and T2) reduces the cell survival, while expression of the long transcripts (T3–T5) has no effect on cell viability. This finding is in concordance with functional duality of PTP4A3, which is reported to regulate both cell survival and metastasis. Given only the expression of short transcripts correlates with cell survival, we can speculate that long transcripts might be involved in cell motility and invasion. This hypothesis requires further exploration.

In summary, we identified and characterized lncRNA ASTILCS, which regulates HCC cell survival presumably via activation of PTK2 mRNA expression and induction of apoptosis. In addition, we unveiled the effects of ASTILCS neighboring genes, PTK2, SLC45A4, and PTP4A3, on HCC cell survival. These findings provide valuable information about HCC biology and can advance the development of future HCC treatments.

## Supplementary information

Supplemental Figure 1. Schematic workflow of shRNA library design.

Supplemental Figure 2. Survival of HCC patients with high and low levels of ASTILCS expression.

Supplemental Figure 3. Positions of shRNAs, sgRNAs and LNA gapmers targeting ASTILCS.

Supplemental Figure 4. PTP4A3 expression in HUH7 cells transduced with sgRNAs targeting ASTILCS transcription start site.

Supplemental Figure 5. Waterfall plot of shRNAs present in the final population of HUH7 cells.

Supplemental Figure 6. Cell cycle progression in HUH7 cells treated with LNA gapmers or shRNAs targeting ASTILCS.

Supplemental Figure 7. Apoptosis in HUH7 cells treated with shRNAs targeting ASTILCS.

Supplemental Figure 8. PTP4A3 gene produces 6 transcripts and 2 protein isoforms.

Supplemental Figure 9. Expression of short PTP4A3 transcripts upon LNA gapmer-mediated knockdown of long PTP4A3 transcripts.

Supplemental Figure Legends

Supplemental Table 1. Long non-coding RNA transcripts expressed in HUH7 cells.

Supplemental Table 2. shRNAs used for validation of the screen results

Supplemental Table 3. sgRNAs used in the study

Supplemental Table 4. LNAs used in the study

Supplemental Table 5. Expression (in FPKM) of genes in the ASTILCS locus in HUH7 cells.

Supplemental Table 6. Population doubling time (Td) for HUH7 cells expressing GFP and ASTILCS.

Supplemental Table 7. SYBR Green primers used in the study

## Data Availability

The sequence data has been submitted to the Gene Expression Omnibus under superseries identifier GSE152651 which consists of the RNA-Seq data (GSE152650) and the shRNA screen data (GSE152649). Original data and numbers for tables are uploaded to Mendeley Data (10.17632/dggchs5s8m.1).
