# Supplementary figures and images for: Identification of a long non-coding RNA regulator of liver carcinoma cell survival
Source: Cell Death Dis. 2021 Feb 15;12(2):178. doi: 10.1038/s41419-021-03453-w (PMC7884843; doi:10.1038/s41419-021-03453-w)

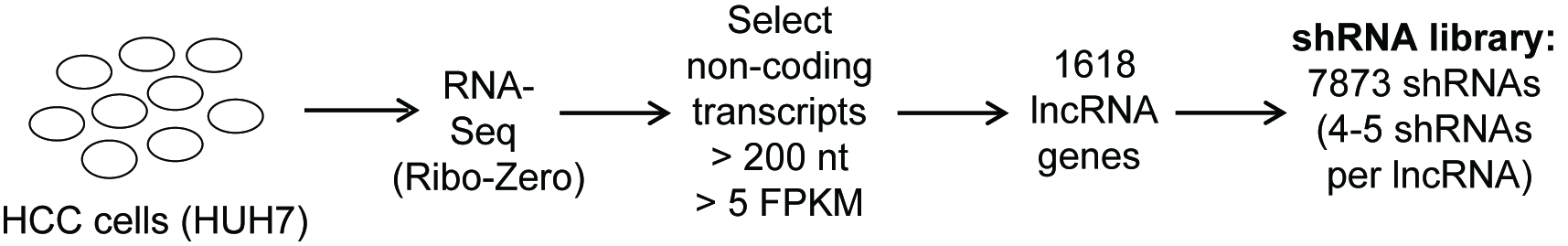

Supplement: Supplementary file 1 — Supplemental Figure 1. Schematic workflow of shRNA library design. [file 41419_2021_3453_MOESM1_ESM.tif]

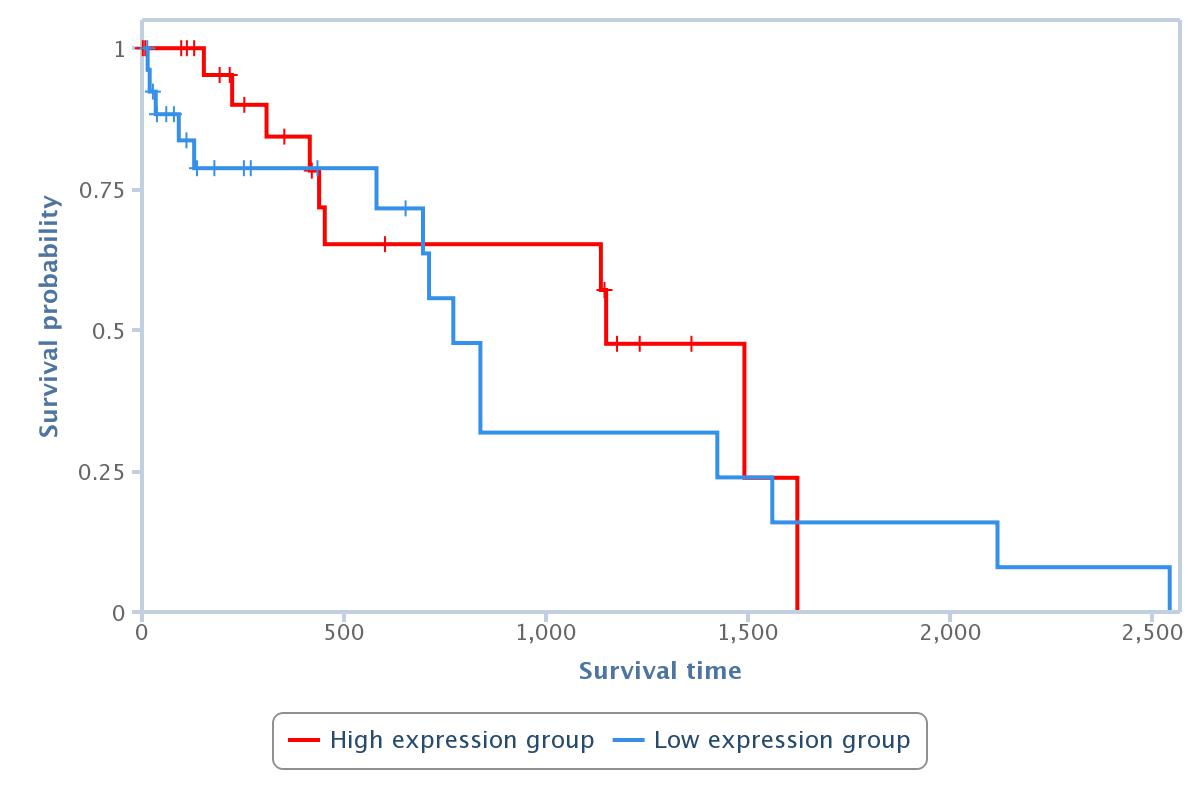

Supplement: Supplementary file 2 — Supplemental Figure 2. Survival of HCC patients with high and low levels of ASTILCS expression. [file 41419_2021_3453_MOESM2_ESM.jpg]

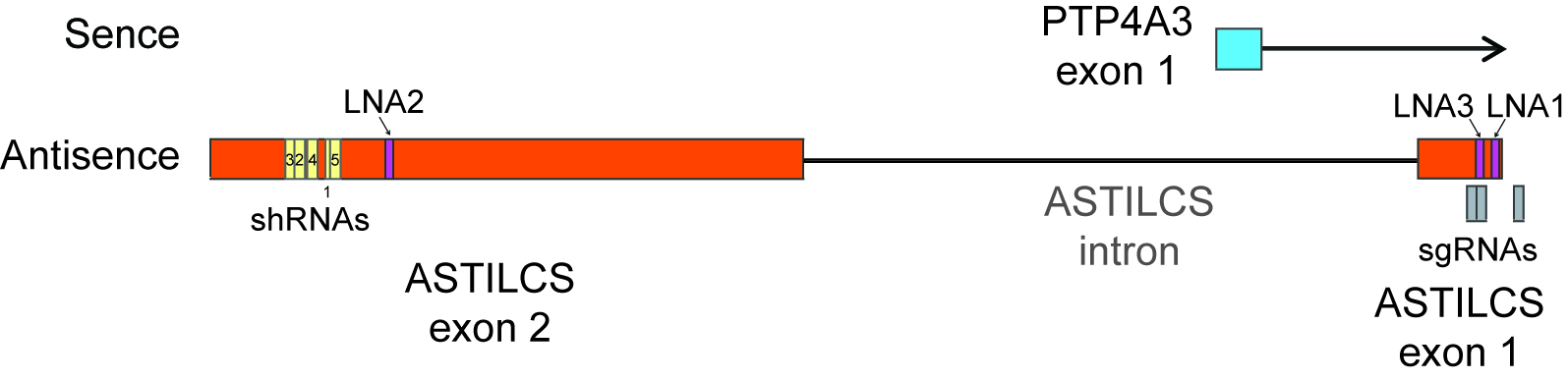

Supplement: Supplementary file 3 — Supplemental Figure 3. Positions of shRNAs, sgRNAs and LNA gapmers targeting ASTILCS. [file 41419_2021_3453_MOESM3_ESM.tif]

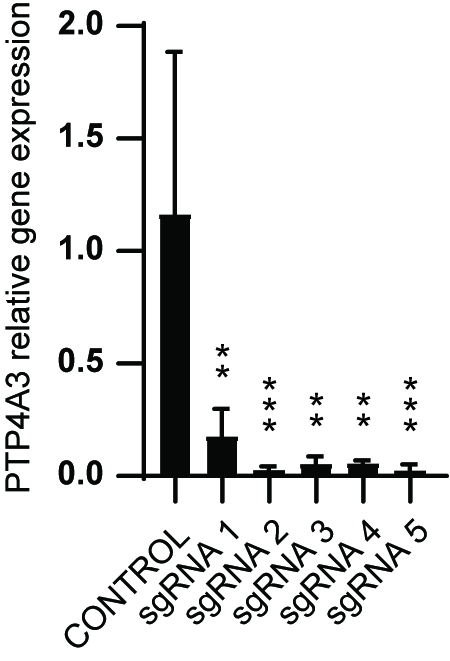

Supplement: Supplementary file 4 — Supplemental Figure 4. PTP4A3 expression in HUH7 cells transduced with sgRNAs targeting ASTILCS transcription start site. [file 41419_2021_3453_MOESM4_ESM.tif]

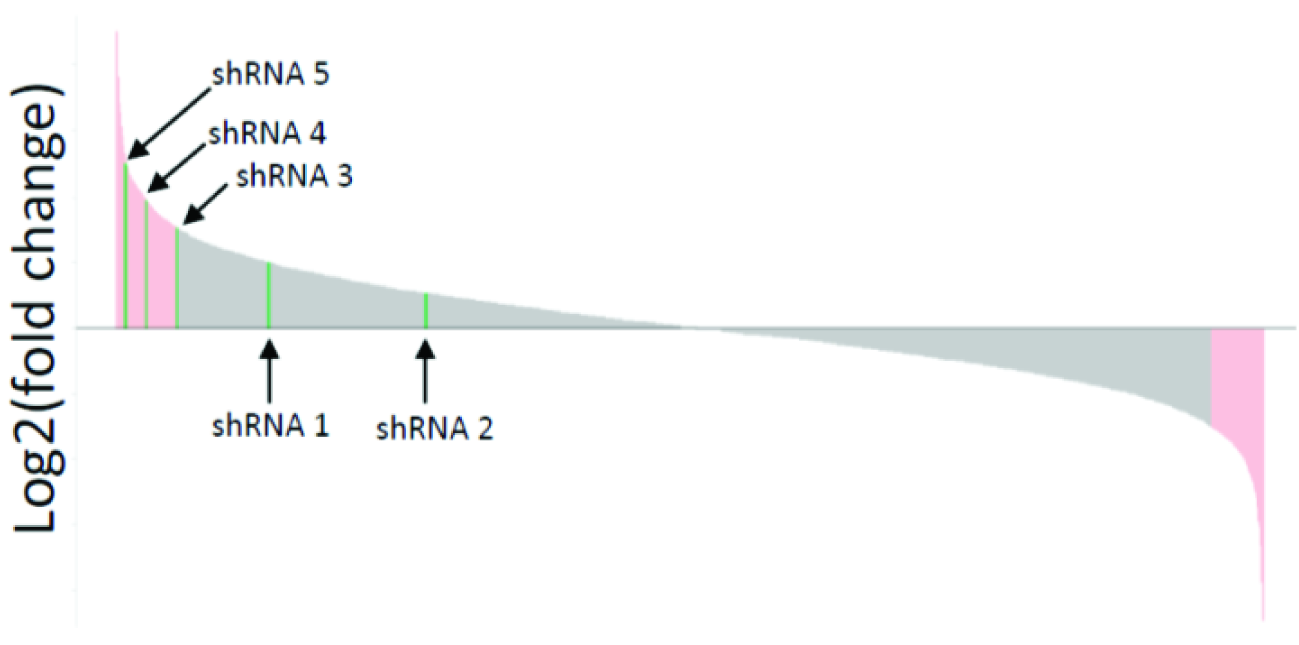

Supplement: Supplementary file 5 — Supplemental Figure 5. Waterfall plot of shRNAs present in the final population of HUH7 cells. [file 41419_2021_3453_MOESM5_ESM.tif]

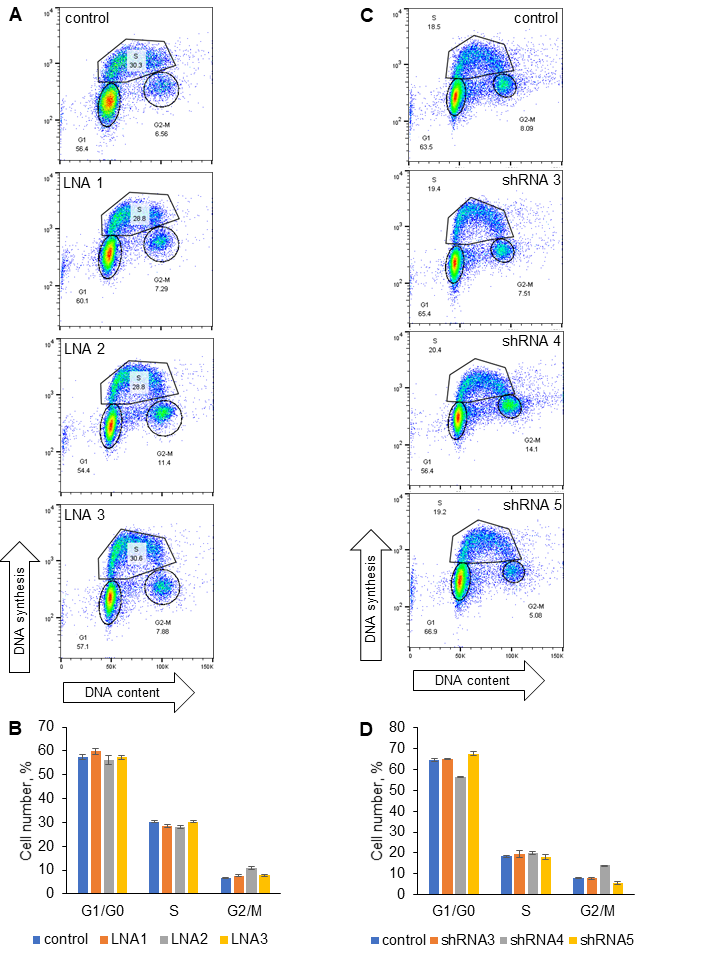

Supplement: Supplementary file 6 — Supplemental Figure 6. Cell cycle progression in HUH7 cells treated with LNA gapmers or shRNAs targeting ASTILCS. [file 41419_2021_3453_MOESM6_ESM.tif]

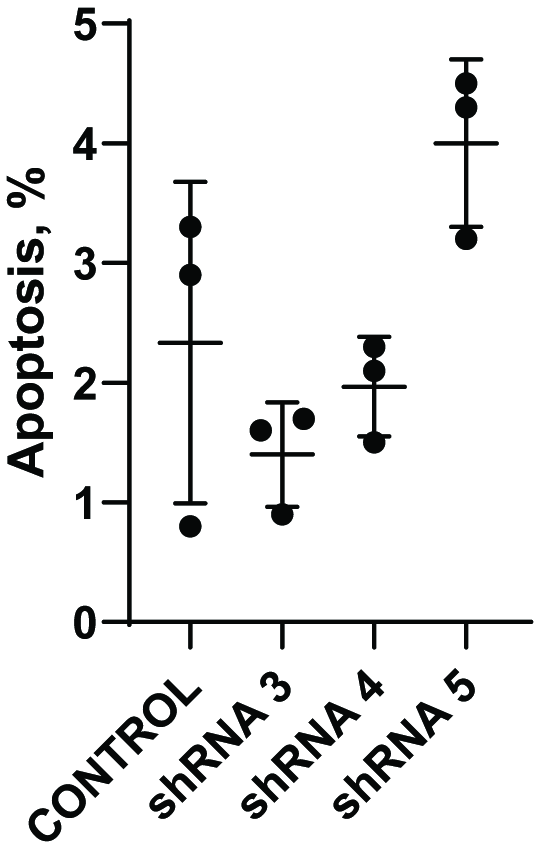

Supplement: Supplementary file 7 — Supplemental Figure 7. Apoptosis in HUH7 cells treated with shRNAs targeting ASTILCS. [file 41419_2021_3453_MOESM7_ESM.tif]

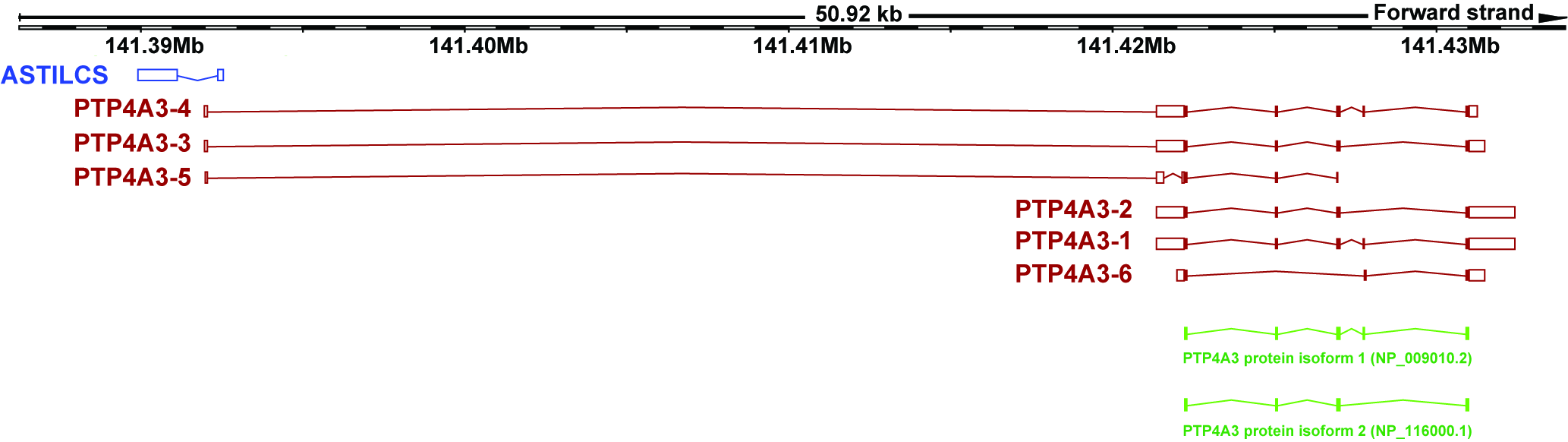

Supplement: Supplementary file 8 — Supplemental Figure 8. PTP4A3 gene produces 6 transcripts and 2 protein isoforms. [file 41419_2021_3453_MOESM8_ESM.tif]

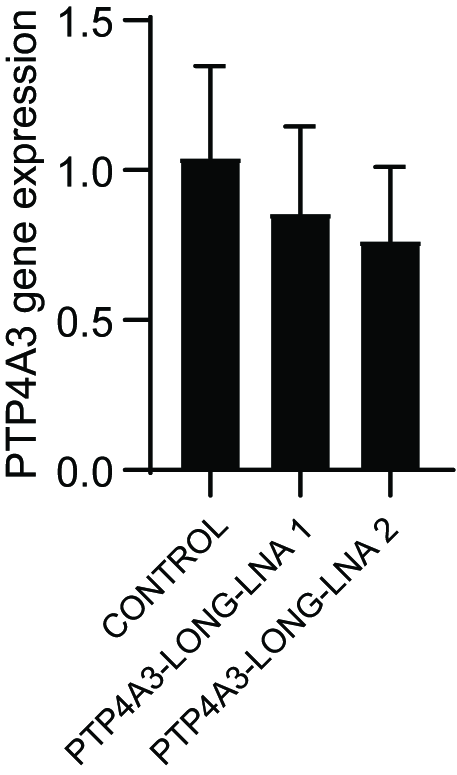

Supplement: Supplementary file 9 — Supplemental Figure 9. Expression of short PTP4A3 transcripts upon LNA gapmer-mediated knockdown of long PTP4A3 transcripts. [file 41419_2021_3453_MOESM9_ESM.tif]
