# Supplementary material for: Identification of a long non-coding RNA regulator of liver carcinoma cell survival
Source: Cell Death Dis. 2021 Feb 15;12(2):178. doi: 10.1038/s41419-021-03453-w (PMC7884843; doi:10.1038/s41419-021-03453-w)
Supplement: Supplementary file 10 — Supplemental Figure Legends [file 41419_2021_3453_MOESM10_ESM.docx]

***Supplemental Figure 1. Schematic workflow of shRNA library design.***

***Supplemental Figure 2. Survival of HCC patients with high and low levels of ASTILCS expression.*** *Long-rank p-value = 0.5*

***Supplemental Figure 3. Positions of shRNAs, sgRNAs and LNAs targeting ASTILCS.***

***Supplemental Figure 4. PTP4A3 expression in HUH7 cells transduced with sgRNAs targeting ASTILCS transcription start site.*** *All values are mean ± SD, n=12, **** p < 0.0001*

***Supplemental Figure 5. Waterfall plot of shRNAs present in the final population of HUH7 cells.*** *Log2(fold change) > or < 0.75 is highlighted in light pink, shRNAs targeting ASTILCS are highlighted in green.*

***Supplemental Figure 6. Cell cycle progression in HUH7 cells treated with LNA gapmers or shRNAs targeting ASTILCS. A, C.*** *Representative dual parameter dot plots of Click-iT® EdU Alexa Fluor® 488 (DNA synthesis) and FxCycle™ Violet (DNA content) showing cell distribution among cell cycle phases.* ***B, D.*** *Percentage of cell cycle phases in control and treated samples. All values are mean ± SD, n=3****.***

***Supplemental Figure 7. Apoptosis in HUH7 cells treated with shRNAs targeting ASTILCS****, All values are mean ± SD, n=3****,*** *there is no significant difference compared to control.*

***Supplemental Figure 8. PTP4A3 gene produces 6 transcripts and 2 protein isoforms.*** *Adapted from* <http://www.ensembl.org/>.

***Supplemental Figure 9. Expression of short PTP4A3 transcripts upon LNA-mediated knockdown of long PTP4A3 transcripts.*** *All values are mean ± SD, n≥8, there is no significant difference compared to control.*
