# Supplementary material for: Identification of a long non-coding RNA regulator of liver carcinoma cell survival
Source: Cell Death Dis. 2021 Feb 15;12(2):178. doi: 10.1038/s41419-021-03453-w (PMC7884843; doi:10.1038/s41419-021-03453-w)
Supplement: Supplementary file 12 — Supplemental Table 2. shRNAs used for validation of the screen results [file 41419_2021_3453_MOESM12_ESM.docx]

***Supplemental Table 2. shRNAs used for validation of the screen results.***

| **shRNA ID** | | **Target sequence** |
| --- | --- | --- |
| Negative control | shRNA 1 | ACTACGACGCTGAGGTCAAGA |
|  | shRNA 2 | GACTACTTGAAGCTGTCCTTC |
| ENST00000429829 | shRNA 1 | GCTCTTCTTTCACGCTTTATT |
|  | shRNA 2 | TCTTTGTGAACTGTGATTATT |
|  | shRNA 3 | TCATGTAATCTCTCCTTAAAT |
|  | shRNA 4 | TCTTAGACATATCTCTCATTT |
|  | shRNA 5 | ATCTCTTGCTGTTTGTGATTT |
| ASTILCS | shRNA 1 | CACAGTGACTCACACTATAAT |
|  | shRNA 2 | AGACCAGCCTAGGTAACATA |
|  | shRNA 3 | GTGGGACCCTATCTCTACAAA |
|  | shRNA 4 | GGATCACTTGAGCCTAGGAAT |
|  | shRNA 5 | ACACTATAATCCCAGCAATTT |
| ENST00000518090 | shRNA 1 | TAACCAAATCACCTCACTGTC |
|  | shRNA 2 | CTTGCCTTGGCCTCCCAATAT |
|  | shRNA 3 | CTCAAATTCCTGGCCTCAAAC |
|  | shRNA 4 | ATGCTGGGATTACAGGCATG |
|  | shRNA 5 | CCTCACTGTCTCTCAAGAGAT |
| ENST00000510145 | shRNA 1 | CCTAGTGAGATGAACCCGGT |
|  | shRNA 2 | CTTTGACTCGGAAAGGGAACT |
|  | shRNA 3 | ACTTTCCAGGTGCCGTCCATC |
|  | shRNA 4 | TGCAGAAATCACCAGTCTTCT |
|  | shRNA 5 | TTCCCGAGTGAGGCAATGCCT |
| ENST00000366097.2 | shRNA 1 | CCAAGTAGTTGGGATTATAGG |
|  | shRNA 2 | GAACTCCTGATCTCAGGTGAT |
|  | shRNA 3 | TTATAGGCGCTTGCCACCATG |
|  | shRNA 4 | GTTGGGATTATAGGCGCTTGC |
| ENST00000457084 | shRNA 1 | ATTGGGAAAGTTGACATTAAT |
|  | shRNA 2 | CTCATTATTCCTCACAGATTT |
|  | shRNA 3 | CTCATCTGAGCCTGGGCAAAT |
|  | shRNA 4 | ATCCAGGTCCTTCTCAGAGAA |
|  | shRNA 5 | TCGCGCAGAAGCTCCTCAATG |
| ENST00000421703.5 | shRNA 1 | GGAACTTTATATTGCCATTTA |
|  | shRNA 2 | GGACCGATATTCTCCAGATTG |
|  | shRNA 3 | TGCTTGAGCCCAGGAGTTTGA |
|  | shRNA 4 | CAGCCTGGGCAACATGGCAA |
|  | shRNA 5 | GCCATTTAGAGGACCGATATT |
