# Supplementary material for: Identification of a long non-coding RNA regulator of liver carcinoma cell survival
Source: Cell Death Dis. 2021 Feb 15;12(2):178. doi: 10.1038/s41419-021-03453-w (PMC7884843; doi:10.1038/s41419-021-03453-w)
Supplement: Supplementary file 13 — Supplemental Table 3. sgRNAs used in the study [file 41419_2021_3453_MOESM13_ESM.docx]

***Supplemental Table 3. sgRNAs used in the study.***

| **sgRNA ID** | **Target sequence** |
| --- | --- |
| control sgRNA 1 | TGTCAGAATTGCAATCTTTG |
| control sgRNA 2 | TTGCAATTCTGACATCTTAT |
| ASTILCS: |  |
| sgRNA 1 | CGGGTCGTAGATGTCAGTGG |
| sgRNA 2 | GGGCGGGTCGTAGATGTCAG |
| sgRNA 3 | GGTCTGAGGCGGACTCCACC |
| sgRNA 4 | CTGACATCTACGACCCGCCC |
| sgRNA 5 | CACAGCCTTCCGTGCCTCCA |
| ENST00000518090: |  |
| sgRNA 1 | ACTGCCTACGAAAGCTGACC |
| sgRNA 2 | ACAGTGAGGTGATTTGGTTA |
| sgRNA 3 | TGAGAGACAGTGAGGTGATT |
| sgRNA 4 | ATTTGGTTAAGGACAATTTC |
| sgRNA 5 | CAATTTCTGGTTCACATTCC |
| ENST00000366097.2: |  |
| sgRNA 1 | GGAACTAGAAAGAAAGCACG |
| sgRNA 2 | AGCACGAGGACCAGCCAGCT |
| sgRNA 3 | GAGCAGGTGCTCCACAGACC |
| sgRNA 4 | AAGCACGAGGACCAGCCAGC |
| sgRNA 5 | GCTGGGAGAGGCCAGGTCTG |
| ENST00000421703.5: |  |
| sgRNA 1 | TATATCGATTCCTAACTTGG |
| sgRNA 2 | AGATGGAAGGGAAGCCAACG |
| sgRNA 3 | AACCACCCAGGGTTCCCCGT |
| sgRNA 4 | TTAGATGGAAGGGAAGCCAA |
| sgRNA 5 | CTATATCGATTCCTAACTTG |
