# Supplementary material for: Identification of a long non-coding RNA regulator of liver carcinoma cell survival
Source: Cell Death Dis. 2021 Feb 15;12(2):178. doi: 10.1038/s41419-021-03453-w (PMC7884843; doi:10.1038/s41419-021-03453-w)
Supplement: Supplementary file 14 — Supplemental Table 4. LNAs used in the study [file 41419_2021_3453_MOESM14_ESM.docx]

***Supplemental Table 4. LNAs used in the study.***

| ID | Qiagen cat. Number | Sequence |
| --- | --- | --- |
| control LNA | LG00000002 | AACACGTCTATACGC |
| ASTILCS - LNA 1 | LG00193623 | GGAAAGCAGAGCGTCA |
| ASTILCS - LNA 2 | LG00193624 | CGGCAATAGAAGCATT |
| ASTILCS - LNA 3 | LG00193625 | AGGGCGGGTCGTAGAT |
| SLC45A4-LNA 1 | LG00230726 | GAGGCGTCGTGGAAGA |
| SLC45A4-LNA 2 | LG00230727 | GCCGTTAAGGAAAAGT |
| PTK2-LNA 1 | LG00230753 | CCGAGTTAGCGGAATA |
| PTK2-LNA 2 | LG00230754 | TCGTCATAAGGCTGTA |
| PTP4A3-SHORT LNA 1 | LG00230746 | CCTTAGCCATCTGTCG |
| PTP4A3-SHORT LNA 2 | LG00230747 | TGAGAAGCTGCCAAAT |
| PTP4A3-LONG LNA 1 | LG00225103 | CAGGATTTGGTTAAGC |
| PTP4A3-LONG LNA 2 | LG00225104 | ATGGATGCGCTCGGTA |
