# Supplementary material for: Identification of a long non-coding RNA regulator of liver carcinoma cell survival
Source: Cell Death Dis. 2021 Feb 15;12(2):178. doi: 10.1038/s41419-021-03453-w (PMC7884843; doi:10.1038/s41419-021-03453-w)
Supplement: Supplementary file 15 — Supplemental Table 5. Expression (in FPKM) of genes in the ASTILCS locus in HUH7 cells. [file 41419_2021_3453_MOESM15_ESM.docx]

***Supplemental Table 5. Expression (in FPKM) of genes in the ASTILCS locus in HUH7 cells.***

|  | **AGO2** | **CHRAC1** | **DENND3** | **GPR20** | **MROH5** | **PTK2** | **SLC45A4** | **TRAPPC9** | **TSNARE1** | **ASTILCS** |
| --- | --- | --- | --- | --- | --- | --- | --- | --- | --- | --- |
| Replicate 1 | 9.98 | 28 | 1.99 | 0 | 0 | 49.04 | 13.27 | 3.99 | 0.49 | 22.26 |
| Replicate 2 | 10.41 | 28.1 | 1.52 | 0 | 0 | 63.81 | 15.21 | 3.87 | 0.31 | 28.39 |
| Replicate 3 | 8.89 | 23.14 | 1.92 | 0 | 0 | 58.63 | 11.54 | 3.24 | 0.32 | 23.27 |
| Replicate 4 | 7.29 | 20.25 | 1.08 | 0 | 0 | 60.3 | 12.58 | 2.6 | 0.35 | 20.06 |
