# Supplementary material for: Identification of a long non-coding RNA regulator of liver carcinoma cell survival
Source: Cell Death Dis. 2021 Feb 15;12(2):178. doi: 10.1038/s41419-021-03453-w (PMC7884843; doi:10.1038/s41419-021-03453-w)
Supplement: Supplementary file 16 — Supplemental Table 6. Population doubling time (Td) for HUH7 cells expressing GFP and ASTILCS. [file 41419_2021_3453_MOESM16_ESM.docx]

***Supplemental Table 6. Population doubling time (Td) for HUH7 cells expressing GFP and ASTILCS.*** *Td was calculated from the exponential portion of the cell growth curve (days 3-5) using the following equation: Td = 0.693t/ln(Nt/N0), where t—time (in days), N0—initial cell number, Nt—cell number on day t.*

|  | **control** | **ASTILCS** |
| --- | --- | --- |
| replicate 1 | 1.17 | 1.16 |
| replicate 2 | 1.07 | 1.13 |
| replicate 3 | 1.02 | 1.10 |
| replicate 4 | 1.02 | 1.11 |
| average | 1.07 | 1.13 |
| Stand. Dev. | 0.07 | 0.03 |
|  | p=0.1698 | |
