# Supplementary material for: Identification of a long non-coding RNA regulator of liver carcinoma cell survival
Source: Cell Death Dis. 2021 Feb 15;12(2):178. doi: 10.1038/s41419-021-03453-w (PMC7884843; doi:10.1038/s41419-021-03453-w)
Supplement: Supplementary file 17 — Supplemental Table 7. SYBR Green primers used in the study [file 41419_2021_3453_MOESM17_ESM.docx]

***Supplemental Table 7. SYBR Green primers used in the study.***

| **Gene ID** | **Forward primer sequence** | **Reverse primer sequence** | **PrimerBank ID** [126], [127] |
| --- | --- | --- | --- |
| ACTB | CATGTACGTTGCTATCCAGGC | CTCCTTAATGTCACGCACGAT | 4501885a1 |
| AGO2 | ACCCGCATCATCTTCTACCG | CTTGTCCCCCGCTCGTT | - |
| ASTILCS | TGCTTCTATTGCCGGGAAGTT | TAAAATGCAGCCACAGTGAAACG | - |
| CHRAC1 | TCGTGGGTAAAGACAAGGGC | TGGCTAGGCATTGAACAAAGAG | 342360617c1 |
| DENND3 | CCCATCCTGTCGGACCAGAT | GGACTTGGAGTAGGTGATGCT | 50345869c3 |
| ENST00000366097.2 | TGGAGATCCAGCCATTACACA | AGTGTCCTTAAAGGGGAGGGG | - |
| ENST00000421703.5 | TGGGGCAATTCCTATGGCTC | CTGTGACGGTTCCCAGAAGT | - |
| ENST00000518090 | GCTGTGCACATCGAGAGAAG | AGGCCCATCGGGTGTATTG | - |
| PTK2 | TGGTGCAATGGAGCGAGTATT | CAGTGAACCTCCTCTGACCG | 313851041c2 |
| PTP4A3 | ACACATGCGCTTCCTCATCA | TCAATGAAGGTGCTGAGCGT | - |
| PTP4A3-LONG | CCTCCACCCGTCGTGC | CCCACTCCATGAACCCCAG | - |
| PTP4A3-SHORT | TGCCCTGTCCTGTCCTGATA | CACAGTCCCAAGAACCGTCA | - |
| SLC45A4 | GCTGTCCCGTCCAAAGACC | GCAGACCCAATGAGAGGTGTG | 122937258c1 |
| TRAPPC9 | TCCTCTACATCCGCTACAGGC | TGATGAGGCCCACGACTTTG | 238624121c1 |
| TSNARE1 | CCCCTAGAGTGCGCTAGATGT | GCCCTTGGGACAATAGGCG | 254750703c1 |
